# Intratumoral Microbiome in Head and Neck Paragangliomas

**DOI:** 10.3390/ijms25179180

**Published:** 2024-08-23

**Authors:** Maria Fedorova, Anastasiya Snezhkina, Dmitry Kalinin, Elena Pudova, Margarita Lantsova, George Krasnov, Vladislav Pavlov, Anna Kudryavtseva

**Affiliations:** 1 Engelhardt Institute of Molecular Biology, Russian Academy of Sciences, 119991 Moscow, Russia; fedorowams@yandex.ru (M.F.); pudova_elena@inbox.ru (E.P.); lantsovams@gmail.com (M.L.); gskrasnov@mail.ru (G.K.); vladislav1pavlov@gmail.com (V.P.); rhizamoeba@mail.ru (A.K.); 2 Vishnevsky Institute of Surgery, Ministry of Health of the Russian Federation, 117997 Moscow, Russia; dmitry.v.kalinin@gmail.com

**Keywords:** head and neck paragangliomas, 16S rRNA gene sequencing, whole transcriptome sequencing, microbiome, bacteria, viruses, negative controls

## Abstract

Head and neck paragangliomas (HNPGLs) are rare neoplasms arising from paraganglia of the parasympathetic nervous system. HNPGLs are characterized by high vascularity and are located in proximity to major vessels and nerves, which may be potential sources of microbial invasion in these tumors. There have been no studies in the literature on the microbiota in HNPGLs. Investigation of the microbiome associated with paragangliomas is important for understanding tumor pathogenesis. In this study, we investigated the microbiome composition in two sets of HNPGLs. First, 29 fresh frozen (FF) tissues were subjected to 16S rRNA gene sequencing; concurrently, a panel of candidate laboratory-derived contaminants was investigated. Second, we analyzed microbial reads from whole transcriptome sequencing data obtained for 82 formalin-fixed paraffin-embedded (FFPE) HNPGLs. The bacterial diversity in FF tumors was found to be significantly lower than that observed in FFPE HNPGLs. Based on 16S rRNA gene sequencing, only seven bacterial families were identified as potential tumor inhabitants: *Bryobacteraceae*, *Enterococcaceae*, *Neisseriaceae*, *Legionellaceae*, *Vibrionaceae*, *Obscuribacteraceae*, and *Mycobacteriaceae*. However, RNA-Seq demonstrated higher sensitivity for identifying microbiome composition and revealed abundant bacterial families that partially correlated with those previously described in pheochromocytomas and extra-adrenal paragangliomas. No viruses were found in HNPGLs. In summary, our findings indicated the presence of a microbiome in HNPGLs, comprising a number of bacterial families that overlap with those observed in pheochromocytomas/paragangliomas and glioblastomas.

## 1. Introduction

Head and neck paragangliomas (HNPGLs) are rare neuroendocrine tumors derived from extra-adrenal parasympathetic paraganglia. HNPGLs account for only 0.03% of autonomic nervous system tumors, whereas 90% of such tumors arise from large adrenal sympathetic paraganglia and are called pheochromocytomas (PCCs) [1]. HNPGLs are characterized by their site of origin: carotid (60%), middle ear (29%), and vagal paragangliomas (13%) [2]. HNPGLs are associated with vascular and neural structures, including the carotid artery, jugular bulb, and cranial nerves, which present a challenge in surgical management. The signs and symptoms of HNPGLs may range from an asymptomatic neck mass to hearing loss, facial and tongue paralysis, and compression of the brain and/or brainstem [3]. These tumors are highly vascularized, slow-growing, and have variable potential for metastasis (2–19%) [2,4].

Significant advances have been made in the genetics and molecular biology of paragangliomas and pheochromocytomas (PPGLs) over the past few decades. A list of genes that predispose carriers to hereditary tumor development has been identified, including *VHL*, *SDHx*, *SDHAF2*, *RET*, *NF1*, *THEM127*, *MAX*, *HIF2A*, *HRAS*, *KIF1B*, *PHD1/2*, *FH*, *SLC25A11*, *MEN1*, *MDH2*, *DLST*, *TP53*, *DNMT3A*, and *GOT2* [5,6]. Furthermore, some somatic alterations in various genes, such as *SDHD*, *SDHA*, *TP53*, *HIF2A*, *KIF1B*, *RET*, *NF1*, *HRAS*, *CSDE1*, *MAML3*, *IDH1/2*, and *TERT*, have been found [6,7]. The Cancer Genome Atlas (TCGA) consortia conducted a comprehensive study on PPGLs and classified them into three molecular subtypes based on mRNA expression patterns [8]. The study also identified subtype-specific molecular alterations, as well as associated genetic and epigenetic changes. However, many questions remain regarding the mechanisms of initiation and progression of PPGLs, particularly sporadic tumors. In addition, HNPGLs have been less researched than PCCs due to their rarity, but they pose a higher risk of metastasis and multifocal tumor development; they also present significant challenges for diagnostics and treatment. This determines the relevance and importance of molecular genetic studies of these tumors.

The presence of a particular microbiome composition in the human body is vital for human health and plays an important role in a variety of physiological functions. A shift in microbiome composition has been observed in many pathologies, and the association of the human microbiome with disease development is becoming clear [9]. Only a few bacteria and viruses have been recognized as directly carcinogenic by the International Agency for Research on Cancer (IARC), including *Heliobacter pylori*, *Epstein–Barr virus*, *hepatitis B virus*, *hepatitis C virus*, *Kaposi’s sarcoma-associated herpesvirus*, *human immunodeficiency virus type 1*, *human papillomaviruses* (12 types), and *human T-cell lymphotropic virus type 1* [10]. However, many microorganisms can indirectly cause cancer [11]. The gut microbiota has been shown to be closely associated with cancer, immunity, and antitumor therapy [12]. However, recent studies have found the existence of multiple microorganisms as a component of the tumor microenvironment that can promote chronic inflammation and induce DNA mutations and the activation of oncogenic molecular pathways contributing to tumor development [13]. Intratumoral microbiota could originate from several potential sources, including mucosal organs through the disruption of mucosal barriers, the circulatory system (hematogenous spread from different body sites), and normal adjacent tissues [14]. Another possible route of microbial distribution is neural transport, which leads to invasion of the nervous system and may be the source of the intratumoral microbiome in the brain. The vagus nerve relays multiple signals from the gut microbiota to the brain and is involved in the direct microbiota–gut–brain axis [15]. A number of studies have demonstrated that bacteria and viruses can gain access to the brain via the vagus nerve, as well as retrograde axonal transport via the trigeminal, olfactory, and facial nerves [16,17,18]. In addition, although the brain has traditionally been considered an organ within a sterile environment, recent research has reported evidence of the presence of microbiota in the normal human brain and in brain diseases [19,20]. Nejman et al. revealed the intratumoral microbiome composition in glioblastoma using a comprehensive combination of molecular methods [21]. A more recent study by Zhao et al. provided evidence for the presence of mostly intracellular bacteria in gliomas based on the visualization of bacterial lipopolysaccharide using a three-dimensional (3D), quantitative in situ imaging strategy [22]. Thus, the intratumoral microbiome is present in tumors of the nervous system and may play a role in their pathogenesis and treatment.

HNPGLs are directly connected to vessels and nerves; carotid paragangliomas are located at the carotid artery bifurcation, whereas vagal paragangliomas occur along the vagus nerve. The anatomical location of these tumors, in conjunction with their high vascularization, determines the probability of microbial invasion and the presence of a distinct microbiome composition. In this study, we analyzed the intratumoral microbiome in HNPGLs using 16S rRNA gene sequencing and microbial reads extracted from RNA-Seq data. Given that solid tumor tissues are always samples with a low microbial biomass, we employed a series of negative controls to minimize the effect of contamination. This study represents a pioneering effort to investigate the microbiome in HNPGLs, which is a crucial step in characterizing these tumors and elucidating their pathogenic mechanisms.

## 2. Results

### 2.1. Microbiome Composition in Fresh Frozen (FF) HNPGL Tissues

**Figure 1:**
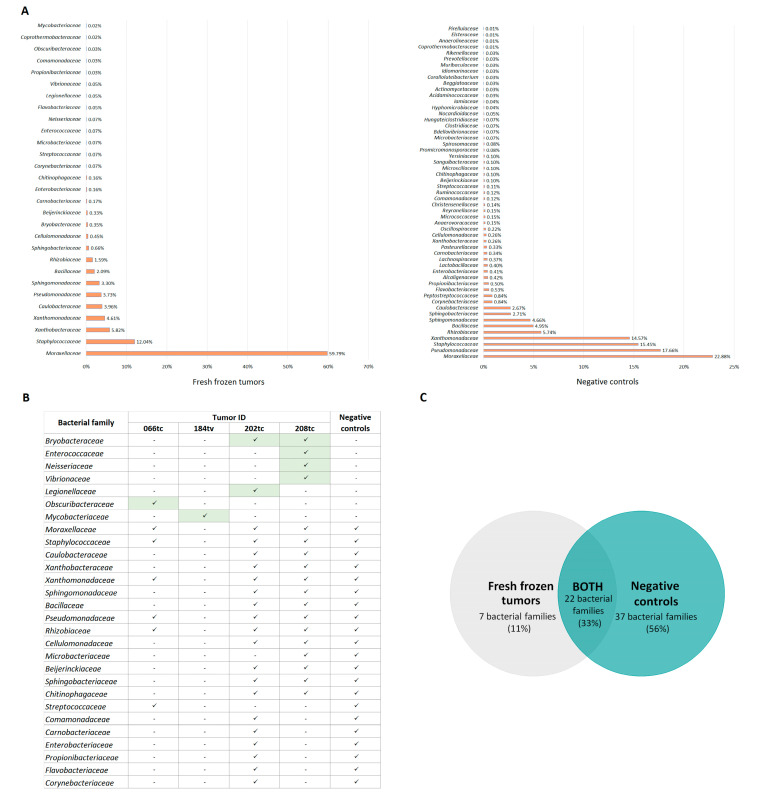
The profile of bacterial families in fresh frozen HNPGLs and negative controls. (**A**) Relative abundance of bacterial families in fresh frozen tissues and negative controls. (**B**) Tumor samples containing bacterial families potentially present in HNPGLs. Green color indicates bacterial families found in tumor samples that were not detected in negative controls. (**C**) Venn diagram showing the number of common and exclusive bacterial families present in fresh frozen tumors and negative controls.

Using 16S rRNA gene sequencing, we evaluated the abundance of bacteria at the family level in 29 fresh frozen surgical specimens of HNPGLs and 156 negative controls that were obtained from possible sources of bacterial contamination in the laboratory. Bacterial sequences were detected in 10 tumors and 37 negative controls. The identified bacterial families and their relative abundance in tumor tissues and negative controls are shown in Figure 1.

The predominance of contaminating bacterial DNA was expected due to the low microbial biomass in the tumor samples. The families *Moraxellaceae*, *Pseudomonadaceae*, *Staphylococcaceae*, *Xanthomonadaceae*, *Rhizobiaceae*, *Bacillaceae*, *Sphingomonadaceae*, *Caulobacteraceae*, and *Xanthobacteraceae* were the major contributors to contamination in FF HNPGLs (Figure 1). Only four tumors were characterized by the presence of a microbiome (*Bryobacteraceae*, *Enterococcaceae*, *Neisseriaceae*, *Legionellaceae*, *Vibrionaceae*, *Obscuribacteraceae*, and *Mycobacteriaceae*) that were not detected in the negative controls, but all had a low quantity of bacterial sequences (Figure 1). These tumors were carotid and vagal paragangliomas and were not specifically associated with *SDHx* mutations. *Mycobacteriaceae* was found in one tumor (184tv) that had no other bacteria. *Enterococcaceae* and *Neisseriaceae* were previously identified as contaminants [23]. Four other bacterial families were detected in tumors with high levels of contamination, suggesting that these bacteria may also be the result of contamination. This is also supported by the higher Shannon diversity index of the negative controls (10.73) compared to the tumor samples (4.73). The list of contaminants found in the negative control samples is shown in Table 1.

### 2.2. Microbiome Composition in Formalin-Fixed Paraffin-Embedded (FFPE) HNPGL Tissues

**Figure 2:**
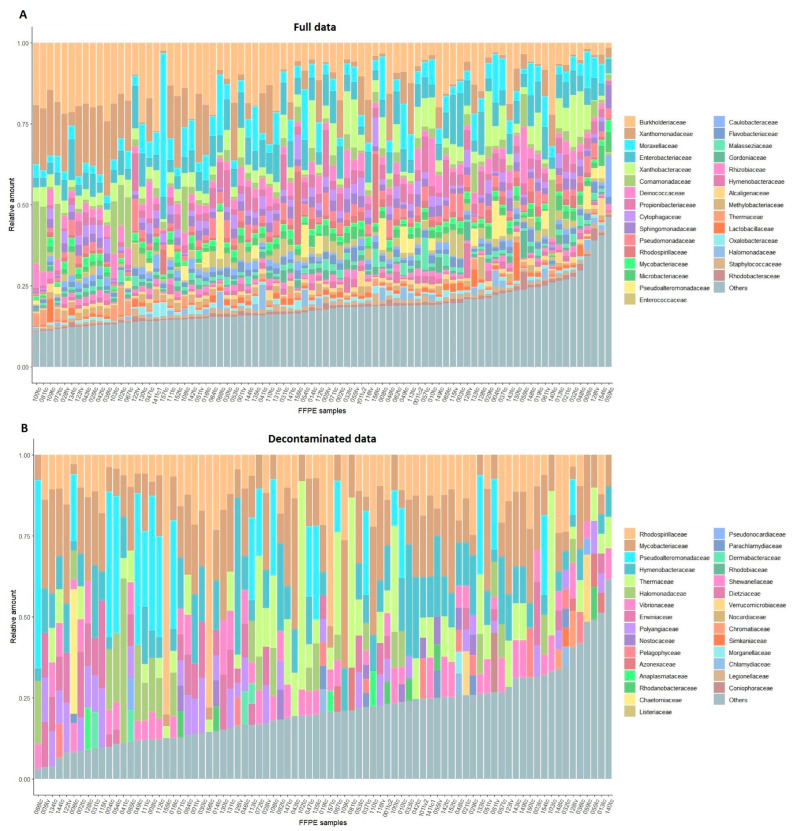
Stacked barplot of the microbiome composition (top 30 expressed families) in FFPE HNPGLs. (**A**) All microbial families detected in HNPGLs (top 30 from full data). (**B**) The top 30 families in the decontaminated data.

A microbiome profiling analysis was conducted on 82 FFPE HNPGLs using whole transcriptome sequencing data. These data were generated based on an rRNA depletion method that allows for the detection of all coding and non-coding transcripts present in the sample. In total, 352 bacteria and fungi were found at the family level in FFPE tumors using the MaxiKraken2 database. The identified families were filtered with a “blacklist” of known contaminants and contaminating bacterial sequences detected in the study. Additionally, the decontam package’s “frequency” method was utilized to identify possible contaminants. Approximately one-third of the identified families were related to contamination (98 out of 352). The Shannon index was 45.45, indicating high microbiota diversity in FFPE tumors. The microbiome composition (top 30 families) across HNPGL samples is shown in Figure 2. A comprehensive list of bacterial and fungal families identified in FFPE HNPGLs is provided in Supplementary File S1. In addition, we analyzed correlations of microbiome composition with clinical and pathological characteristics (tumor location and features, sex, age, *SDHx* mutations) using Spearman’s correlation and the Mann–Whitney test for subgroups with FDR correction. No associations were found.

Virome analysis revealed the presence of virus contigs in multiple samples. Upon meticulous examination, it was established that all identified viruses were either bacteriophages or viruses with other potential contaminating microorganisms as hosts. Subsequent alignment using the blastn and blast nt databases in the Blast Web Service excluded all remaining viral contigs, as they were mapped to other organisms with a greater length and E-value (Supplementary File S1).

## 3. Discussion

A multitude of microorganisms, including bacteria, viruses, fungi, archaea, and protists, are commonly found in various human organs, including epithelial surfaces, the oral cavity, and the gastrointestinal, respiratory, and urogenital tracts [24]. The number of studies investigating the association between the gut microbiota and cancer is rapidly increasing. Dysbiosis of the gut microbiota can promote tumorigenesis and alter the response to chemotherapy and immunotherapy. Conversely, certain gut microbes (e.g., *Lactobacillus*, *Bifidobacterium*, *Faecalibaculum rodentium*, and *Streptococcus thermophiles*) have been demonstrated to possess anticarcinogenic properties [25]. The presence of microorganisms within solid tumors has been identified through histological and genomic methods, providing evidence of a distinctive microbiome composition in each type of tumor [21,26]. Although the precise role of the intratumoral microbiome in different cancers remains to be fully elucidated, recent studies have demonstrated its involvement in tumorigenesis and metastasis, as well as its association with the efficacy of anticancer therapy. Finally, in 2022, the polymorphic microbiome was discussed as a potential new hallmark of cancer [27].

This study addresses the intratumoral microbiome composition in HNPGLs. To date, only a few studies have specifically analyzed the microbiome in neuroendocrine neoplasms and brain tumors that are closely related to HNPGLs in terms of origin or localization [21,22,28,29]. Massironi et al. employed the fluorescent in situ hybridization (FISH) method to investigate the presence of bacteria in pancreatic and intestinal neuroendocrine neoplasms (NENs) [28]. The researchers observed a high degree of bacterial infiltration in both pancreatic tumors and NENs, with rates of 90% and 75%, respectively. However, the taxonomic composition of these bacteria has not been studied in detail. More recently, it has been demonstrated that bacteria are present in NENs of the pituitary gland [29]. Nejman et al. conducted a comprehensive investigation of the microbiome in several solid tumor types, including glioblastoma [21]. The intratumoral microbiome composition in glioblastoma was assessed based on 16S rRNA gene sequencing data, with rigorous control for potential contaminations. The microbiome composition in glioblastoma was identified to comprise 12 bacterial families, including *Microbacteriaceae*, *Micrococcaceae*, *Exiguobacteraceae*, *Planococcaceae*, *Rhizobiaceae*, *Neisseriaceae*, *Enterobacteriaceae*, *Moraxellaceae*, *Xanthomonadaceae*, *Staphylococcaceae*, *Rhodobacteraceae*, and *Pasteurellaceae*. The 16S rRNA gene sequence screening of FF HNPGLs samples identified the presence of almost all of the aforementioned families, with the exception of *Exiguobacteraceae*, *Planococcaceae*, and *Rhodobacteraceae*. The analysis of RNA-Seq data for FFPE HNPGLs revealed the presence of all families, except *Exiguobacteraceae*. In general, the 16S rRNA gene sequencing results indicated a limited microbial diversity in FF HNPGLs. A limited number of bacterial families have been identified as potentially associated with tumors: *Bryobacteraceae*, *Enterococcaceae*, *Neisseriaceae*, *Legionellaceae*, *Vibrionaceae*, *Obscuribacteraceae*, and *Mycobacteriaceae*. Nevertheless, due to the low number of bacterial reads per family, the high contamination level of tumor samples, and the possibility of these families being contaminants as previously reported, it is necessary to consider the potential sterility of HNPGLs.

In contrast, the analysis of whole transcriptome sequencing data revealed a more diverse microbiome in FFPE samples, characterized by a Shannon index of 45.5, which is approximately ten-times higher than that obtained for FF tumors (4.73). This discrepancy can be attributed to the greater depth and sensitivity of the transcriptome sequencing method, as well as the potential for sequencing batch effects, which can result in the generation of excessive false positive results. Moreover, RNA-Seq encompasses not only bacterial sequences but also those of other microorganisms. Furthermore, FFPE tumor samples present technical challenges for study and may be more susceptible to microbial contamination than FF samples [30]. As part of a large-scale microbiome study based on whole exome and transcriptome sequencing data from the TCGA project for 33 types of tumors [26], a similar analysis was conducted for PPGLs. The study identified 94 taxa, including bacteria, archaea, and viruses, in PPGLs under the most stringent decontamination (http://cancermicrobiome.ucsd.edu/CancerMicrobiome_DataBrowser/ accessed 20 June 2024). Our study identified cross-links between PPGLs and HNPGLs in 36 bacterial families. However, 14 of these families were marked as contaminants. Notably, no viruses were identified in HNPGLs, while PPGLs exhibited a significant viral burden (29 taxa). The observed differences in microbial composition between PPGLs and HNPGLs may be attributed to a number of factors, including the type of samples (TCGA—FF, our dataset—FFPE), multiple technical factors (RNA extraction, library preparation, and data analysis), the specificity of the studied cohort (the TCGA PPGL cohort consists mainly of PCCs and extra-adrenal PGL tumors), and the effectiveness of removing true contaminants. In a related study, Chen and colleagues conducted an analysis of the microbiome for the TCGA PPGL cohort based on transcriptome sequencing data, particularly miRNA-Seq [31]. The bacterial composition identified in PPGLs (the top 15 taxa) is available in the BIC database (http://140.112.52.86:8888/bic/analyses/composition/ accessed 20 June 2024). All of the top 15 bacterial families detected in PPGLs were also found in HNPGLs. Among these, seven families were present in the top 15 list for HNPGLs: *Burkholderiaceae*, *Moraxellaceae*, *Enterobacteriaceae*, *Comamonadaceae*, *Sphingomonadaceae*, *Pseudomonadaceae*, and *Rhodospirillaceae*. Thus, the bacterial composition observed in PPGLs is highly analogous to that observed in HNPGLs. However, numerous bacterial families that are commonly found in PPGLs and HNPGLs were identified as contaminants in our study or were frequently observed in previous studies and included in the “blacklist”. Nevertheless, it is possible that many of these results are false negatives due to decontamination, and that the bacteria in question are actually present in the tumor microenvironment. 

This study is subject to several limitations. The most significant limitations are the type of samples (FFPE samples in RNA-Seq analysis), the size of the sample set (a limited number of FF samples in the 16S rRNA gene sequencing study), and the absence of normal controls. The availability of adjacent normal tissues was limited by the small initial size of parasympathetic paraganglia in the head and neck. Furthermore, the donation of normal paraganglia from deceased donors was also unavailable. Consequently, it was not possible to investigate the presence of microbiota in normal paraganglia and/or the specific microbiome composition associated with paragangliomas. Another significant limitation is the potential for contamination. Despite the implementation of experimental negative controls and the consideration of contaminating taxa reported in the literature, the distinction between true intratumoral inhabitants and contaminants remains an unresolved issue. Finally, the data are limited by the identification of microorganisms at the family level.

## 4. Materials and Methods

### 4.1. Tumor Specimens and Controls

A collection of 29 fresh frozen tumor tissues from patients with HNPGLs was utilized for the 16S rRNA-based metagenomics study. The tumor tissues were procured during surgical procedures and immediately frozen in liquid nitrogen. The frozen tissue samples were stored at −80 °C. RNA-Seq data previously obtained from 82 FFPE HNPGLs were subjected to metatranscriptome analysis. All tumor specimens were obtained from patients who had not received preoperative radiation or chemotherapy. The study was approved by the ethics committee of the Vishnevsky Institute of Surgery and conducted in accordance with the Declaration of Helsinki. Prior to the commencement of the study, written informed consent was obtained from all participants. The clinical and pathological characteristics of the patient cohorts are presented in Table 2.

Several types of negative controls were used to exclude laboratory contamination: 35 controls including samples from different surfaces and equipment in pathology anatomy and molecular genetics laboratories, 111 paraffin controls (paraffin areas without tumor tissue), and 10 controls from DNA extraction (blank), PCR, and clean-up steps (no template).

### 4.2. Nucleic Acid Extraction

FF tumor tissue (20 mg) was mechanically homogenized using a MagNA Lyser Instrument (Roche, Basel, Switzerland). DNA was extracted from FF HNPGLs using a Blood & Cell Culture DNA Kit (Qiagen, Hilden, Germany), followed by microbial DNA enrichment using a NEBNext Microbiome DNA Enrichment Kit (New England Biolabs, Ipswich, MA, USA). DNA from controls was isolated using a DNA-sorb-B kit (AmpliSens, Moscow, Russia). For previously obtained RNA-Seq data, RNA was extracted using a High Pure FFPET RNA Isolation Kit (Roche, Basel, Switzerland). DNA and RNA quantification was performed using a Qubit 2.0 Fluorometer (Thermo Fisher Scientific, Waltham, MA, USA).

### 4.3. 16S rRNA Library Preparation, Sequencing, and Analysis

Amplicon-based 16S rRNA libraries were prepared using primers directed to the V3–V4 variable region of the 16S rRNA gene: forward-CCTACGGGNGGCWGCAG and reverse-GACTACHVGGGTATCTAATCC. Two-step PCR was used to amplify the target region with the addition of Illumina sequencing adapters and sample-specific 8 bp dual-index barcodes. All PCR reactions were performed using a Tersus Plus PCR kit (Evrogen, Moscow, Russia) on a T100 thermal cycler (Bio-Rad, Hercules, CA, USA). The first-stage PCR was performed with the following program: initial denaturation at 95 °C for 3 min, followed by 30–50 cycles (depending on sample type) of denaturation at 95 °C for 30 s, annealing at 55 °C for 30 s, and extension at 72 °C for 30 s, with final extension at 72 °C for 5 min, and hold at 4 °C. PCR products were size-verified by 2% agarose gel electrophoresis (~550 bp) and purified using MagPure A4 XP magnetic beads (Magen, Guangzhou, China). The second-stage PCR amplification program was as follows: 95 °C for 3 min, followed by 8 cycles of 95 °C for 30 s, 55 °C for 30 s, and 72 °C for 30 s, with a final extension at 72 °C for 5 min, and hold at 4 °C. PCR amplicons were purified using magnetic beads. The size of the final libraries was examined on an Agilent 2100 Bioanalyzer (Agilent Technologies, Santa Clara, CA, USA) and was ~630 bp. The libraries were then mixed equimolarly and diluted to 10pM loading concentration with 40% of the control library PhiX (Illumina, San Diego, CA, USA). Sequencing was performed on an Illumina MiSeq system using a 300 × 2 bp paired-end run.

Quality control of the raw sequence data was performed using FastQC (https://www.bioinformatics.babraham.ac.uk/projects/fastqc/ accessed 20 June 2024). Reads were trimmed using Trimmomatic v0.38 [32] and were merged into a single fragment using MeFiT v1.0 [33]. Merged reads were filtered based on the Q25 score, and the V3–V4 region of the 16S rRNA gene (438–467 bp) was extracted. To purify reads from human gDNA admixture, the resulting sequences were additionally aligned to the human reference genome (GRCh38, Ensembl release 104) using bowtie2 [34] and STAR [35] in consecutive order. The sequences were then analyzed using the DADA2 Bioconductor package v1.32.0 [36]. Identified ribosomal sequence variants were annotated using the RDP naive Bayesian classifier (DADA2 package) and the Silva v138.1 database with a confidence threshold of 0.8. Based on the obtained data, bacterial taxonomic classification was performed for each sample.

### 4.4. RNA-Seq Analysis

Metatranscriptomic analysis was performed using previously obtained RNA-Seq data for HNPGLs. Transcriptome libraries were prepared using a TruSeq Stranded Total RNA Library Prep Kit with Ribo-Zero Human/Mouse/Rat (Illumina). Human cytoplasmic rRNA was removed from total RNA samples, which were further processed for cDNA library construction. The resulting libraries were sequenced on an Illumina NextSeq 500 system using a 76 bp single-end run.

Raw sequencing reads were qualified using FastQC and then trimmed using Trimmomatic v0.38. A search for bacterial sequences was performed in each tumor sample. RNA-Seq data were purified from human DNA and RNA by aligning sequences to the human reference genome (GRCh38, Ensembl release 104) using bowtie2. Sequences were then aligned using STAR in a two-step process, including detection of novel splice junctions. Sequences that did not map anywhere on the human reference genome were analyzed using Kraken 2 [37]. Taxonomic assignment of metagenomic sequences was performed using the MaxiKraken2 database (140 GB) with a confidence threshold of 0.00 due to short reads. The decontam package v1.24.0 [38] was utilized for the statistical identification of possible contaminants.

Virome analysis was performed based on a pipeline described by Liu et al. [39]. RNA-Seq reads were purified as described above. The remaining non-host reads were assembled de novo using MEGAHIT v.1.2.9 [40]. The coverage of each contig was calculated using BBMap v.39.06 [41]. The assembled contigs were first aligned against viral nucleotides and proteins using blastn and blastx (BLAST v.2.15.0 [42]) with an E-value cutoff of 1E-2. These contigs were then compared with the blast nt database (downloaded in February 2024) using blastn (E-value cutoff at 1 × 10^−10^) to eliminate false positives (non-viral contigs with >60% identity and >10% query coverage).

### 4.5. Statistical Analysis

Statistical analysis was performed using the R Statistical Software v3.2.0. The Shannon diversity index was calculated to estimate the diversity of species within each category of the dataset. The parameter is a measure of both species richness and evenness.

## 5. Conclusions

This study demonstrated the existence of an intratumoral microbiome in HNPGLs, which is predominantly bacterial. No evidence was found to suggest the presence of viruses. Furthermore, the use of whole transcriptome sequencing (rRNA depletion method) demonstrated a higher sensitivity for microbial detection compared to 16S rRNA gene sequencing. Furthermore, a list of potential laboratory contaminants was generated, including several bacterial families reported as such for the first time. This list can be used to reduce the impact of contamination in further similar studies of samples with low microbial biomass.

In-depth characterization of the intratumoral microbiome in HNPGLs may prove beneficial in comprehending the biological mechanisms underlying tumor formation and aggressiveness. It is crucial to investigate the metabolic and signaling functions of distinct microbiota composition and their impact on the tumor immune microenvironment and molecular pathways in tumor cells. This may facilitate the identification of novel targets for anticancer therapy and biomarkers.

## Figures and Tables

**Table 1:** The list of contaminant families found in the negative control samples.

Family *	Negative Control Sample
***Beggiatoaceae***, ***Coralloluteibacterium***, ***Coprothermobacteraceae***, ***Anaerolineaceae***, ***Elsteraceae***, *Pirellulaceae*	Paraffin
*Staphylococcaceae*, *Carnobacteriaceae*, ***Xanthobacteraceae***	Paraffin, reagents
*Enterobacteriaceae*, ***Cellulomonadaceae***, ***Promicromonosporaceae***	Reagents
*Flavobacteriaceae*, *Propionibacteriaceae*, *Lactobacillaceae*, *Lachnospiraceae*, *Pasteurellaceae*, *Oscillospiraceae*, ***Anaerovoracaceae***, *Micrococcaceae*, ***Christensenellaceae***, *Comamonadaceae*, *Streptococcaceae*, *Beijerinckiaceae*, *Chitinophagaceae*, ***Sanguibacteraceae***, ***Yersiniaceae***, ***Spirosomaceae***, *Microbacteriaceae*, ***Bdellovibrionaceae***, *Clostridiaceae*, *Nocardioidaceae*, *Hyphomicrobiaceae*, ***Iamiaceae***, *Acidaminococcaceae*, *Actinomycetaceae*, ***Idiomarinaceae***, ***Muribaculaceae***, *Prevotellaceae*, *Rikenellaceae*	Surface swab
***Microscillaceae***, ***Hungateiclostridiaceae***, *Moraxellaceae*, *Bacillaceae*, *Sphingomonadaceae*, *Sphingobacteriaceae*, *Caulobacteraceae*, *Alcaligenaceae*, ***Reyranellaceae***, ***Ruminococcaceae***	Surface swab, paraffin
*Pseudomonadaceae*, *Xanthomonadaceae*, *Rhizobiaceae*	Surface swab, reagents, paraffin

* Bacterial families first identified as laboratory contaminants are shown in boldface type.

**Table 2:** Clinical and pathological characteristics of patients with HNPGLs.

Characteristic	Number of Patients, n	
	Transcriptome Study	16S Amplicon-Based Study
Total patients	79	29
Total number of tumors	82	29
Sex		
Male	24	8
Female	55	21
Age at diagnosis		
≥40	30	8
<40	49	21
Mean	47.5	48.9
Tumor localization		
Carotid paragangliomas	69	22
Vagal paragangliomas	13	7
Tumor feature		
Single	72	26
Bilateral/multiple	7	3
Recurrent	6	1
Metastasis	3	0
Mutation		
*SDHB*	12	3
*SDHC*	5	1
*SDHD*	24	11
Unknown	0	2

## Data Availability

All data generated or analyzed during this study are included in this published article. The 16S rRNA gene-targeted sequencing and transcriptome sequencing data are available in the NCBI SRA under the accession number PRJNA993587.

## Supplementary Materials

The following supporting information can be downloaded at: https://www.mdpi.com/article/10.3390/ijms25179180/s1.

## Abbreviations

HNPGLs, head and neck paragangliomas. PCCs, pheochromocytomas. PPGLs, paragangliomas and pheochromocytomas. TCGA, The Cancer Genome Atlas. IARC, International Agency for Research on Cancer. FF, fresh frozen. FFPE, formalin-fixed paraffin-embedded. FISH, fluorescence in situ hybridization. NENs, intestinal neuroendocrine neoplasms.
